# Fabrication of Stable Carbon Nanotube Cold Cathode Electron Emitters with Post-Growth Electrical Aging

**DOI:** 10.3390/mi9120648

**Published:** 2018-12-07

**Authors:** Jung Hyun Kim, Jung Su Kang, Kyu Chang Park

**Affiliations:** Department of Information Display and Advanced Display Research Center, Kyung Hee University, Dongdaemun-gu, Seoul 02447, Korea; wjdgus791@khu.ac.kr (J.H.K.); kangjungsu@khu.ac.kr (J.S.K.)

**Keywords:** carbon nanotube, field emitters, electrical aging, Joule heating, electron emission

## Abstract

We fabricated carbon nanotube (CNT) cold cathode emitters with enhanced and stable electron emission properties and long-time stability with electrical aging as a post-treatment. Our CNT field emitters showed improved electrical properties by electrical aging. We set the applied bias for effective electrical aging, with the bias voltage defined at the voltage where Joule heating appeared. At the initial stage of aging, the electron emission current started to increase and then was saturated within 3 h. We understood that 5 h aging time was enough at proper aging bias. If the aging bias is higher, excessive heating damages CNT emitters. With the electrical aging, we obtained improved electron emission current from 3 mA to 6 mA. The current of 6 mA was steadily driven for 9 h.

## 1. Introduction

Carbon nanotubes (CNTs) have attracted much attention in vacuum nanoelectronic applications, especially in the field of cold cathode-based vacuum devices. CNTs have several excellent advantages as cold cathode emitters such as high aspect ratio, high thermal conductivity, and structural rigidity. Many post-treatment processes have been conducted to improve the electrical properties of CNTs [1,2,3,4,5,6,7]. Because of these various advantages, CNTs are suitable for electron sources. In this paper, we studied the enhancement of electrical properties of CNT emitters for their application as cold cathode emitters. [8,9] The stable and long-term lifetime of a CNT-based cold cathode electron beam (C-beam) is one of the most important requirements for commercialization. We developed the electrical aging techniques to fabricate high-performance CNT cold cathode emitters. It was confirmed that the thermal energy could change the crystallinity of CNTs through thermal annealing at 1000 °C [2,6]. In addition, the improved crystallinity could enhance the electrical characteristics, such as lower turn-on field and better stability of electron emission. In a high field emission current, Joule heating by an electron current through CNT emitters and non-uniform electron emission from each CNT emitter was a serious issue to be solved for lifetime enhancement. In previous studies, electrical aging removed the catalyst to induce tip opening and improved the emission current [3]. In this study, we observed the change of the electron emission characteristics by Joule heating. The crystallinity of CNT emitters is one of the most important factors to enhance electron emission. We used this thermal energy by resistive heating on CNT emitters for the structural modification of the CNT emitters. When Joule heating appears during field emission, the temperature of the CNT tip and body increase depending on the electron emission current. While growing CNTs, it is very difficult to control their structure effectively. As the aging time continues, at high currents, only a small amount of the CNT emitter from the non-uniform structure takes up the majority of the emission current, whereas surplus emission current incurs the destruction of these CNTs, which makes the current unstable. In addition, relatively weak CNT emitters are removed by resistive heating during electrical aging at high currents [10,11,12,13,14,15,16,17,18,19,20]. Thus, the aging allowed a relatively larger number, and more evenly distributed shorter multi-walled carbon nanotubes (MWCNTs) to become dominating emitters resulting in improving the field emission (FE) reproducibility [21,22]. According to numerous previous studies, other mechanisms involved in electrical aging are mainly the desorption of adsorbates caused by CNT heating [23,24], changes in geometric structure [25,26,27,28,29,30,31], and particle cleaning. These are the reasons why it is significant to carry out electrical aging to stabilize electron emission [32]. The heat generated by the resistance of the CNT emitter during electron emission could modify the structure of the CNT emitters. The Joule heat improved the crystallinity of the CNT emitters, resulting in stable electron emission. By adopting post-processing technology called electrical aging, we obtained a stable lifetime of 9 h at a 97 mA/cm^2^ current density in a very simple and effective way.

## 2. Materials and Methods

### 2.1. Fabrication of Carbon Nanotube (CNT) Field Emitters

The CNT emitters were grown on a SiO_2_ layer with an *n*-type silicon (Si) substrate, followed by a 30 nm Ni deposition with radio frequency magnetron sputtering. Then, through the photo-lithography process, the diameter of the CNT emitters were designed to be 3 μm and the distance between the emitters was set to 15 μm. Two hundred and twenty-five CNT emitters constituted one island, and a total of 128 (8 × 16) islands were patterned. They were grown by direct current plasma-enhanced chemical vapor deposition for 60 min. The growth temperature was 630 °C, the voltage of graphite susceptor (cathode) was −620 V, and the mesh voltage was + 320 V. Acetylene (C_2_H_2_) and ammonia (NH_3_) gas were fed at rates of 20 sccm and 160 sccm, respectively, during the growth time [33,34]. After the growth, 1000 °C annealing was performed for 1 h in an argon gas atmosphere of 130 mTorr.

### 2.2. Electrical Aging Technology

**Figure 1:**
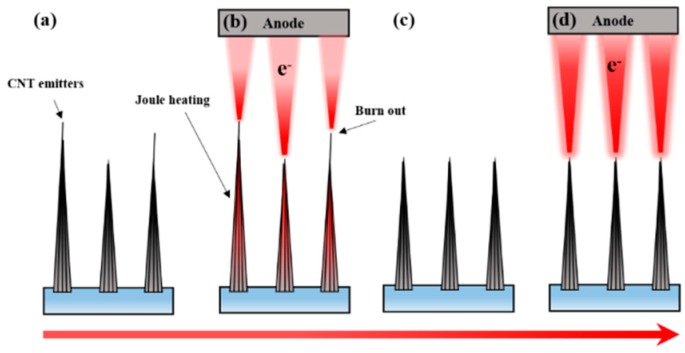
Schematic diagram of electrical aging technology. (**a**) Non-uniformity of height and crystallinity before aging; (**b**) electrical aging with Joule heating; (**c**) homogenization of height and crystallinity; (**d**) stable electron field emission by structural improvement.

Our electrical aging technique follows the schematic diagram of Figure 1. As shown in Figure 1a, the grown CNT emitters have non-uniform height and crystallinity. The uniformity of CNT height is difficult to control with deposition conditions. This structural issue causes a non-uniform field of individual CNT emitters. After various trials to set the aging condition, the starting point of electrical aging was defined as the bias where the Joule heat appeared in the overall emitter area. The Joule heat comes from current flow through the resistive nature of the CNT emitters. Figure 1b shows that we used the thermal energy by Joule heating as a structural modification of the emitters. Figure 1c shows that structurally unstable parts in the CNTs were burned out by the Joule heat and the height of the CNT emitters decreased, resulting in the homogenization of height. The heat also improved the crystallinity of the CNTs. As shown in Figure 1d, the homogenized structure and improved crystallinity formed a uniform field and enhance emission current, enabling stable electron emission characteristics at the higher current level.

**Figure 2:**
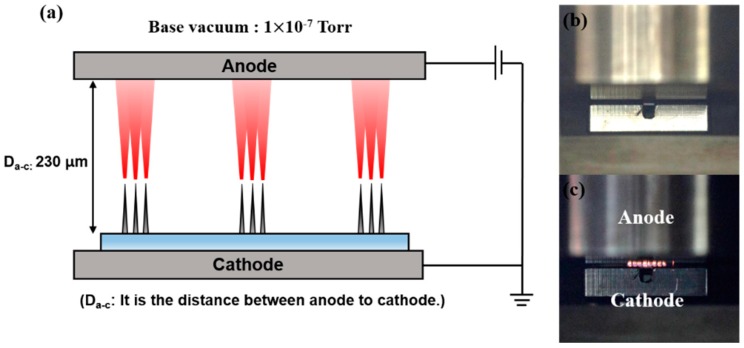
Diode system for measuring current-voltage (I-V) characteristics. (**a**) Schematic diagram of diode system; (**b**) digital single lens reflex camera image of the diode system; (**c**) observed Joule heating when the electron emission current is higher.

This process was performed in a vacuum chamber. Figure 2 shows the schematic diagram and the digital photo image of the diode system used in the experiment. The base vacuum is 1 × 10^−7^ Torr and the distance between the cathode and the anode is 230 μm in the diode system. Figure 2c shows the Joule heating by high electron emission current from the observed CNT emitter. Joule heating images were observed with a digital single lens reflex camera (Canon, EOS-60D, Tokyo, Japan). The electron emission characteristics were measured with a direct current (DC) power supply (Spellman, SL 1200, Suffolk, NY, USA) and a multimeter (Agilent, 3441A, Santa Clara, CA, USA). We analyzed the crystallinity and morphology of the CNT emitters using Raman spectroscopy (Horiba Jobin-Yvon, LabRam ARAMIS, Kyoto, Japan) with a 514 nm excitation laser wavelength, scanning electron microscope (Hitachi, S-4700, Tokyo, Japan). All scanning electron microscope (SEM) images were taken with an accelerating voltage of 10 kV and a 45° tilt.

## 3. Results and Discussion

**Figure 3:**
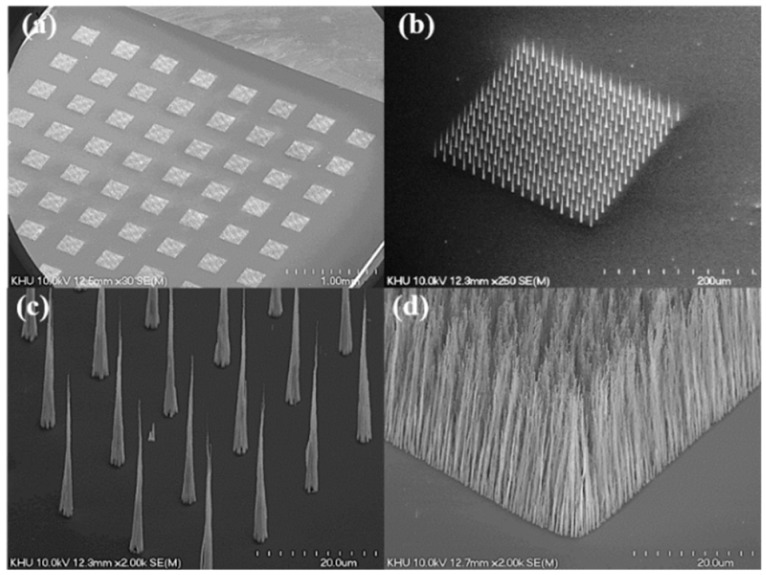
Scanning electron microscope (SEM) images of carbon nanotube (CNT) emitters grown by triode direct-current plasma-enhanced chemical vapor deposition (dc-PECVD). (**a**) Patterned CNT islands image; (**b**) magnified image of one island; (**c**) vertically aligned CNT emitters with cone shape in the island; (**d**) non-patterned CNT.

SEM images of the grown CNT field emitters are shown in Figure 3. As shown in Figure 3a, eight islands are patterned in 16 lines. Figure 3b shows one patterned island SEM image. One island has 15 × 15 array emitters. The CNT emitter is grown vertically on the Si substrate. Figure 3c shows that the single CNT emitter has a diameter of 3 μm and an average height of 40 μm, and unlike the non-patterned emitters in Figure 3d, the patterned CNT emitters have a cone shape and only one CNT at the tip.

**Figure 4:**
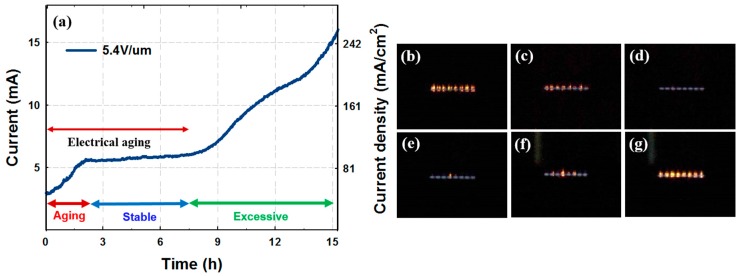
Definition of electrical aging. (**a**) Variation of electron emission current at a higher emission regime with time; (**b**) the section at which Joule heating occurs sufficiently; (**c**) the section at which Joule heating is reduced; (**d**) the section at which Joule heating disappears completely; (**e**) the section at which Joule heating reappears; (**f**) the section at which Joule heating increases; (**g**) excessive Joule heating that can cause damage to the CNT emitters.

Field emission characteristics over time during aging were associated with Joule heating. We categorized the emission currents in relation to Joule heating. The categories we classified are shown in Figure 4. At an emission current of 3 mA, overall Joule heating occurred and this bias was set as the starting point of electrical aging. The eight divided lights clearly show that the Joule heating appears in the emitter area. During 2 h of electrical aging, the emission current increased from 3 mA to 5.5 mA, and after 3 h the Joule heating disappeared completely. After aging was completed, stable electron emission was maintained for 5 h without visible light. The Joule heating began to reappear when the emission current increased, and the CNT emitter was damaged by the excessive Joule heating with increased electron emission current. 

**Figure 5:**
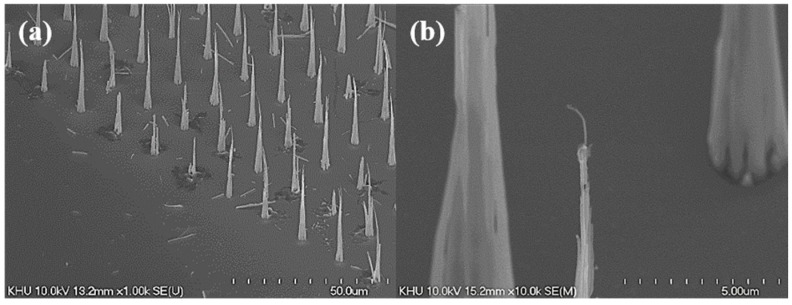
(**a**) Damaged CNT emitters by excessive Joule heating; (**b**) tip of a structurally modified emitter.

SEM images of the CNT emitter damaged by excessive Joule heating are shown in Figure 5. Figure 5a shows a number of broken emitters. Figure 5b shows the tips of one emitter that has been damaged. The bias set position of the electrical aging is very important. Excessive Joule heating causes damage to CNT emitters. However, at an aging current lower than the Joule heat light appeared, the aging effect is insignificant. In order to carry out electrical aging most effectively, we set the bias and current to those at which the Joule heating light appears in the entire emitter region as electrical aging conditions. 

**Figure 6:**
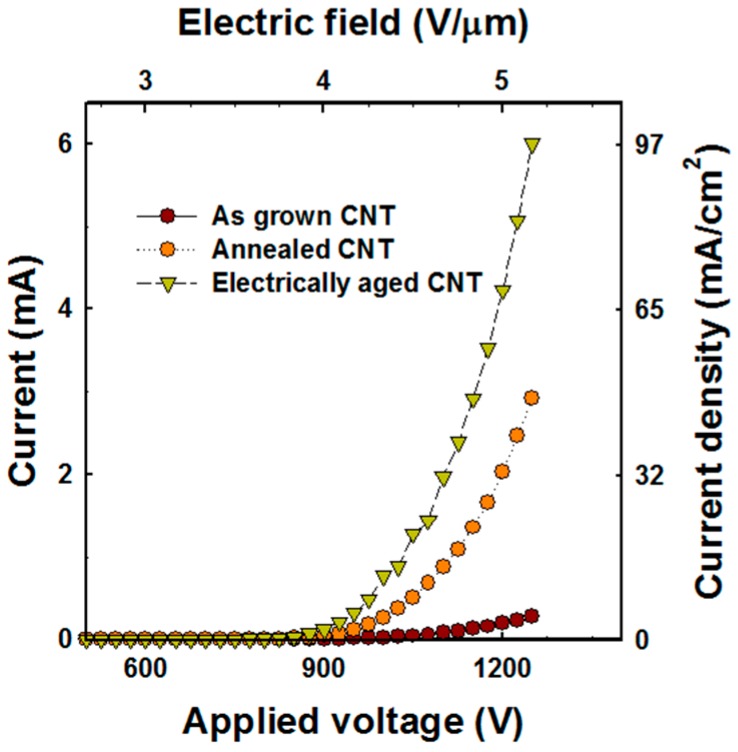
Field emission properties with treatment condition.

We were able to confirm the decrease in the turn-on field and the increase in the emission current density after the post-growth treatment. Current-voltage (I-V) characteristics for each treatment are shown in Figure 6 and summarized in Table 1. The electron emission characteristics could improve after annealing at 1000 °C. When electrical aging was completed, the current density increased to 97 mA/cm^2^ at the same electric field, which is 20 times higher than the current density of as-grown CNTs. It is expected that the aging effect will cause the structural modification of the CNT emitter. So, we compared the structural properties and crystallinity of each treatment by SEM and Raman analysis.

**Figure 7:**
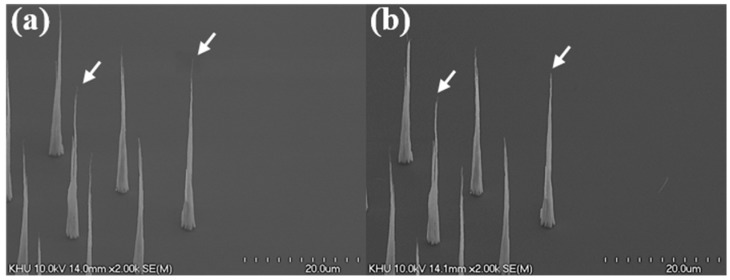
SEM image before and after aging. (**a**) CNT emitters before aging; (**b**) decreased CNT height (white arrow) after aging.

Figure 7 shows an SEM image to compare the structural features of the CNT emitters before and after electrical aging. In Figure 7a,b, the height reduction in the weak area of the CNT emitters were confirmed after electrical aging. A weak and unstable area of the CNT emitter burns out after aging. The uniform height contributes to a uniform electric field formation on CNT emitters. Uniform field formation allows individual nanotubes to emit a uniform amount of current and greatly contributes to improved lifetime.

**Figure 8:**
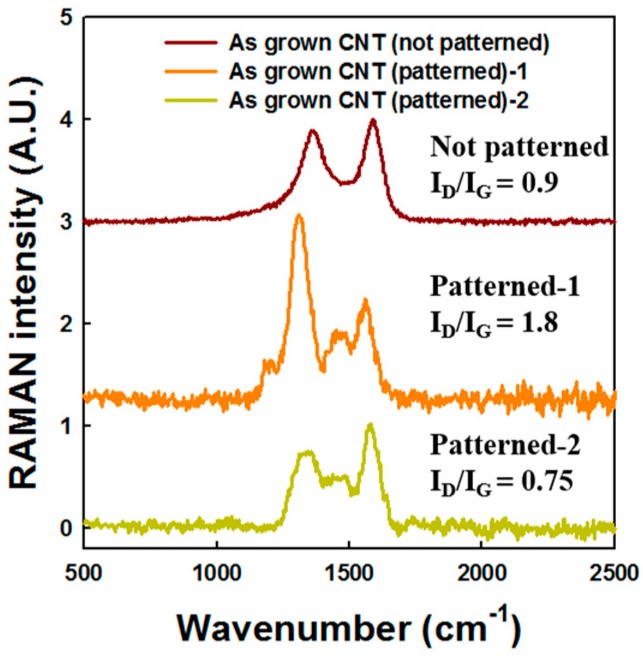
Normalized Raman spectra of patterned and non-patterned as-grown CNT samples. Raman spectra acquired with a 514 nm excitation laser.

Figure 8 shows the Raman spectra of patterned and non-patterned as-grown CNT emitters. A D peak at 1350 cm^–1^, and a G peak at around 1580 cm^–1^ appeared. As shown in Figure 8, the Raman spectrum revealed that non-patterned CNT emitters analyzed the Raman signals with several numbers of CNTs, but in the case of patterned CNT emitters, only one CNT emitter analyzed the Raman signals. The resolution of the Raman spectroscopy used was ~1 μm. The Raman signal of non-patterned CNTs showed an intensity ration of D and G band (I_D_/I_G_) ratio of 0.9 and a clear Raman signal because so many numbers of CNTs were measured. However, the patterned CNTs showed a large distribution of the I_D_/I_G_ ratio. The variation of the G/D ratio is related to the crystallinity of each CNT. To compare the crystalline uniformity of CNT emitters, four CNT measurements were taken and compared to non-patterned CNT emitters with aging via Raman analysis. 

**Figure 9:**
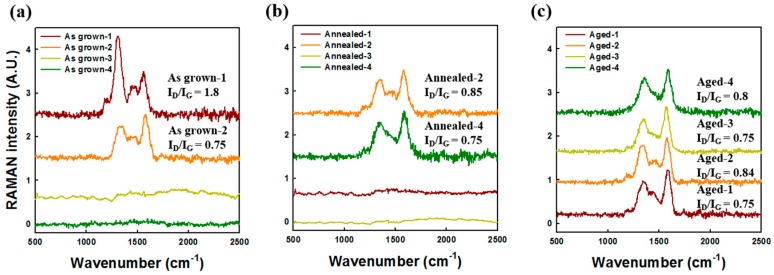
Normalized Raman spectra of patterned (**a**) as-grown, (**b**) annealed, (**c**) and electrically aged CNTs.

Figure 9 shows the Raman spectra of patterned CNT emitters for each treatment. As shown in Figure 9a, four sample emitters were analyzed with Raman measurement. Two out of four samples show no Raman signal. It was found that as-grown samples had non-uniform crystallinity. Figure 9b shows that two Raman signals of 1000 °C annealed CNT were measured, similar to the as-grown patterned CNT emitters. However, the samples show a decrease in the variation of the I_D_/I_G_ ratio. We confirmed the improvement of crystallinity by a thermal energy of 1000 °C annealing. However, the uniformity of the crystalline structure of the CNT emitters was insufficient by 1000 °C annealing. In Figure 9c, the Raman signal appeared for all four samples, and the I_D_/I_G_ ratios were similar in all four signals. From the Raman analysis, it can be seen that the electrical aging contributes to the crystallinity enhancement of the CNT emitter and that a more uniform crystallinity appears with the electrical aging. This uniform structural characteristic would enable a long-term stable field emission.

**Figure 10:**
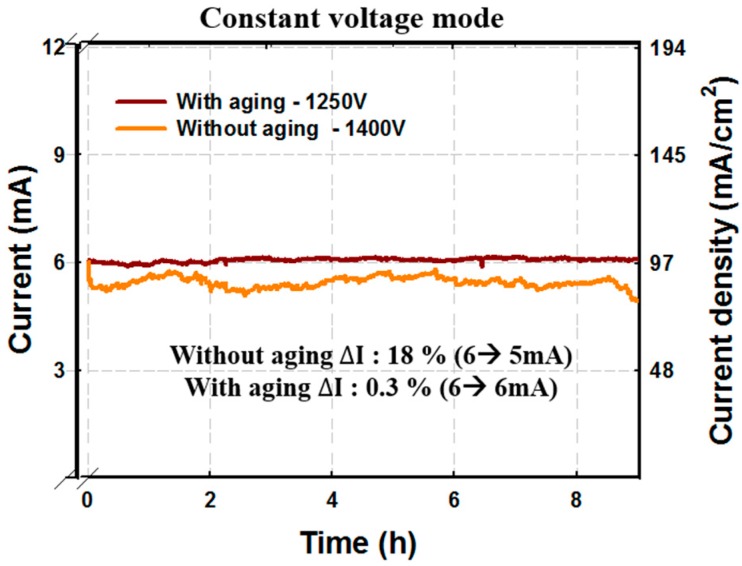
Comparison of stability with and without electric aging. Electrically aged emitters were shown to be more stable.

Figure 10 shows a comparison of the long-term stability of CNT emitters without and with electrical aging. The emission current was measured in constant voltage mode at 30-s steps. CNT emitters without electrical aging showed the degradation of the electron emission current from 6 mA to 5 mA after 9 h. However, for CNT emitters with electrical aging, the electron emission current did not degrade and a very uniform emission current was achieved. The fluctuation of the emission current was reduced by electrical aging to 0.3% (△I = standard deviation/average current × 100). As a result, although the crystalline property of CNTs improved after annealing at 1000 °C, the crystallinity of each sample was not uniform and was insufficient for uniform long-time operation. However, the electrical aging induced uniform structural properties and crystallinity. The uniform and strong structure of electrically aged CNT emitter enabled stable operation even at high current emissions.

## 4. Conclusions

In this study, Joule heating generated at a high current emission on resistive CNT emitters was used to induce the self-annealing of the CNT emitters. With the thermal energy caused by Joule heating applied to the CNT emitters, the structure of individual CNT emitters changed and resulted in uniform emitters. In addition, owing to the elevated temperature during electron emission, we could enhance the crystallinity of the CNT emitters. With the electrical aging technique, structurally and electrically improved CNT emitters were made. We were able to obtain the stable long-term operation of CNT emitters with a current density of 97 mA/cm^2^ for 9 h after electrical aging. If the Joule heating was excessive, the CNT emitters were damaged. To set the electrical aging current, just before the Joule heating light appeared was determined to be the best bias position of the current level. This electrical aging technique represents a simple and a better post-growth treatment process for the achievement of high-performance cold cathode CNT emitters that drive steadily, even at high current emission levels.

## Figures and Tables

**Table 1:** Comparison of electrical properties of carbon nanotube (CNT) emitter with treatment condition. DC–direct current.

Treatment	Driving	Turn on Field (V/μm)	Current Density at 5.4 V/μm (mA/cm^2^)
As-grown		3.6	4.5
Annealed	DC	3.3	48
Electrically aged		3	97
